# “Yiqi Huayu, Wenyang Lishui” Prescription (YHWLP) Improves the Symptoms of Chronic Obstructive Pulmonary Disease-Induced Chronic Pulmonary Heart Disease by Inhibiting the RhoA/ROCK Signaling Pathway

**DOI:** 10.1155/2021/6636426

**Published:** 2021-10-26

**Authors:** Hui Huang, Kuizhong Shan, Min Cai, Hong Chen, Fengmei Wu, Xiaoyan Zhao, Huawei Zhuang, Hong Li, Suofang Shi

**Affiliations:** ^1^Department of Respiratory Diseases, Kunshan Hospital Affiliated to Nanjing University of Chinese Medicine, Kunshan, Suzhou 215300, Jiangsu, China; ^2^Department of Oncology, The Second People's Hospital of Kunshan, Suzhou 215300, Jiangsu, China; ^3^Department of Respiratory Diseases, Jiangsu Province Hospital of TCM, The Affiliated Hospital of Nanjing University of Chinese Medicine, Nanjing 210000, Jiangsu, China

## Abstract

**Background:**

Chronic pulmonary heart disease (CPHD) is a common type of heart disease. In China, chronic obstructive pulmonary disease (COPD) is one of the main causes of CPHD. At present, there is no specific therapy for COPD-induced CPHD, so it is of great importance to identify a new therapy for CPHD.

**Objective:**

The purpose of this study was to explore the effects of “Yiqi Huayu, Wenyang Lishui” prescription (YHWLP) on CPHD symptoms.

**Methods:**

Eighty patients with COPD-induced CPHD were randomly divided into the control group and the YHWLP group, both involving treatment for 3 months. Both groups were treated with Western medicine, and the YHWLP group was also treated with YHWLP. The changes (relative to baseline) in the symptoms, pulmonary arterial pressure, prothrombin time (PT), activated partial thromboplastin time (aPTT), fibrinogen (Fbg), D-dimer (D-D), and ratio of phosphorylated (p)-myosin-binding subunit (MBS)/total (t)-MBS in peripheral blood (which indirectly indicates the activation/inhibition of RhoA/ROCK signaling) were compared between the two groups.

**Results:**

YHWLP plus Western medicine was superior to Western medicine alone at reducing symptoms, pulmonary arterial pressure, PT, aPTT, Fbg, D-D, and p-MBS/t-MBS.

**Conclusion:**

YHWLP can relieve CPHD by inhibiting the RhoA/ROCK signaling pathway, which means YHWLP is a potential treatment for CPHD.

## 1. Introduction

Chronic pulmonary heart disease (CPHD) is a common type of heart disease that is characterized by pulmonary arterial hypertension, which causes right ventricular dysfunction and hypertrophy and eventually leads to chronic heart failure. CPHD may be caused by a variety of factors, including bronchial and pulmonary lesions, nerve, muscle, and chest wall lesions, and an abnormal ventilation drive [1–4]. In China, chronic obstructive pulmonary disease (COPD) is one of the main causes of CPHD, accounting for about 85% of CPHD cases [1, 5, 6]. COPD primarily involves chronic airway inflammation and lung parenchyma destruction. The incidence, morbidity, and mortality rates are increasing year by year [7]. COPD was ranked fifth in terms of economic burden of disease worldwide in 2020, and it is expected to rank third in terms of mortality by 2030. In recent years, CPHD has become a major cause of disability, and the mortality rate associated with COPD-induced CPHD is also increasing year by year [8]. Unfortunately, there is no effective treatment for CPHD. Therefore, it is of great importance to identify a new therapy for CPHD.

In recent years, with the development of research on traditional Chinese medicine (TCM), a growing number of studies have reported the efficacy of TCM for treating lung diseases [9–12]. Compared to Western medicine, TCM has the advantages of fewer adverse reactions and significant curative effects. Studies have shown that a variety of TCM prescriptions can alleviate CPHD symptoms, which has provided a solid foundation for the widespread use of TCM for the clinical treatment of CPHD [13–16]. According to TCM theory, deficiency in vital energy in relation to the lungs can cause a variety of diseases. CPHD patients generally have symptoms of deficiency in vital energy, including obvious fear of cold and cold limbs. Yiqi, Huayu, Wenyang, and Lishui are the four basic TCM principles for the treatment of heart failure, and various TCM prescriptions based on these principles have achieved positive clinical outcomes [14, 17–20]. It is generally recognized that TCM components for Yiqi and Wenyang (such as, cinnamon bark and ginseng) can be used to treat lung diseases. However, it remains unclear whether the “Yiqi Huayu, Wenyang Lishui” prescription (YHWLP) can effectively relieve CPHD symptoms.

Many studies have shown that the RhoA/ROCK signaling pathway plays an important role in the regulation of pulmonary vasoactivity [21, 22]. Research on this pathway has attracted widespread attention. Therefore, this study aimed to explore the clinical effects of YHWLP on COPD-induced CPHD (i.e., the effects on symptoms, pulmonary function indexes, pulmonary hypertension, and coagulation-related indexes) and to assess whether the mechanism involves inhibiting the RhoA/ROCK signaling pathway.

## 2. Materials and Methods

### 2.1. Patients

Eighty patients (48 males and 32 females) with COPD-induced CPHD were enrolled in this study from October 2017 to May 2018 at the Respiratory Department of Kunshan Hospital of Traditional Chinese Medicine. The age range was 52–80 years, with a mean age of 68.42 ± 1.528 years. All patients met the inclusion and exclusion criteria, which included being diagnosed based on both Chinese and Western medicine criteria. The study was approved by the ethics committee of Kunshan Hospital of Traditional Chinese Medicine, and all patients signed an informed consent form. Patients were randomly divided into the control group and YHWLP group. Power analysis was performed by G^*∗*^Power software (version 3.1.9.7, https://www.psychologie.hhu.de/arbeitsgruppen/allgemeine-psychologie-und-arbeitspsychologie), and based on the results, the actual power is greater than 0.8 when the samples size was 40 in each group. So, the sample size was determined as *n* = 40 in each group.

### 2.2. Inclusion and Exclusion Criteria

The inclusion criteria were as follows: (1) patients met the Western medicine diagnostic criteria for COPD-induced CPHD (compensatory stage) based on the Revised Standards of the Second National Conference on Cor Pulmonale [23] (the criteria are mainly based on medical history, physical signs (including pulmonary function), electrocardiography, X-ray results (including radioisotope examinations), echocardiography, and vectorcardiography); (2) TCM syndrome differentiation: “Qixu Luoyu, Yangxu Shuiting” (wheezing (which is particularly severe when moving), cough, expectoration, chest tightness, shortness of breath, spontaneous sweating, catching colds easily, fatigue, edema, cyanosis of the lips, light/dark swollen tongue, weak and bounding pulse, or astringent pulse); (3) signed informed consent form; and (4) able to complete the study.

The exclusion criteria were as follows: (1) lack of CPHD; (2) brain, liver, or kidney disease or other organ failure; (3) psychosis; (4) poor compliance/difficulty cooperating; (5) aged <16 or >85 years; (6) pregnant or lactating; (7) allergic to YHWLP; and (8) TCM syndrome differentiation: not “Qixu Luoyu, Yangxu Shuiting.”

### 2.3. Treatments

The patients were randomly divided into the YHWLP and control groups (40 per group), and treatment lasted for 3 months in both groups. Both groups were treated with Western medicine, and the YHWLP group was also treated with YHWLP (300 ml, double decoction) daily.

The Western medicine treatment plan was based on treatment for CPHD in the remission period according to Internal Medicine (seventh edition published by the People's Health Publishing House). The treatment was based on the principles of controlled oxygen therapy, reducing phlegm, relieving asthma, and ensuring diuresis. The specific treatment plan was as follows: (1) controlling the oxygen concentration (FiO_2_ <35%) and oxygen saturation (85–95%); (2) expectorant: 10 ml mucosultan (tid, oral); (3) antiasthmatic agent: Xinbeike (4.5 *µ*g/160 *µ*g, bid, two inhalations) or Seretide Accuhaler (50 *µ*g/500 *µ*g, bid, one inhalation); and (4) diuretic agent: furosemide (20 mg, qd) or amiloride (one pill, qd).

We also analyzed the components of the concentrated YHWLP decoction and the YHWLP granules using high-performance liquid chromatography. The main components of YHWLP were mulberry glycoside A, paeoniflorin, *Codonopsis* alkynoside, and glycyrrhizic acid. The results are shown in the supplementary materials (available here).

### 2.4. Clinical Symptom Scores

The clinical symptom scores were assessed at baseline and after treatment. The symptoms and signs were scored as follows: (1) The main symptoms and signs (cough, expectoration, wheezing, spontaneous sweating, cold, edema of lower limbs, cyanosis, and lung rales or wheezes) were each scored as normal (0 points), mild (2 points), moderate (4 points), or severe (6 points). (2) The secondary symptoms and signs (sticky mouth, dry mouth, or bitter taste, could not taste salt, full abdominal distension, and dry/loose stools) were each scored as normal (0 points), mild (1 point), moderate (2 points), or severe (3 points). Tongue and pulse conditions were not scored. (3) Efficacy was then assessed as follows: based on the score reduction rate (total posttreatment score−total baseline score)/total baseline score: (a) clinical control: score reduction rate ≥90% (symptoms and signs disappeared or almost disappeared); (b) considerable effect: score reduction rate ≥70% (symptoms and signs were significantly improved); (c) effective: score reduction rate ≥30% (symptoms and signs were improved); and (d) ineffective: score reduction rate <30% (small improvement or worsening of symptoms and signs).

### 2.5. Measurement of Lung Function

Lung function was assessed in terms of forced expiratory volume in the first second (FEV1), forced vital capacity (FVC), FEV1/FVC, and FEV1 as a percentage of the predicted value (FEV1%).

### 2.6. Measurement of Pulmonary Artery Pressure

The mean pulmonary artery pressure was calculated as 0.61 × pulmonary artery systolic pressure (PASP) + 2 mmHg. PASP was calculated as tricuspid regurgitation pressure gradient + right atrial pressure, assessed by color Doppler echocardiography. According to the Bernoulli equation, peak tricuspid regurgitation pressure gradient = 4 × peak tricuspid regurgitation velocity. The right atrial pressure (normally 5–10 mmHg) was estimated based on respiratory variation in the inferior vena cava diameter. This method has a good correlation with hemodynamic data based on right heart catheterization in patients with CPHD.

### 2.7. Measurement of Prothrombin Time (PT), Activated Partial Thromboplastin Time (aPTT), Fibrinogen (Fbg), and D-Dimer (D-D) in Peripheral Blood

No patients took anticoagulants (such as warfarin or aspirin) in the 2 weeks before blood sampling. Blood samples (5 ml) were obtained from the elbow vein in the morning, centrifuged, and then, stored. An automatic coagulometer was used to assess PT, aPTT, and Fbg, along with D-D (via the immunoturbidimetric method), according to the manufacturer's instructions for the assay reagents.

### 2.8. Measurement of Phosphorylated (p)-Myosin-Binding Subunit (MBS)/Total (t)-MBS Ratio in Peripheral Blood

To indirectly assess the activation/inhibition of RhoA/ROCK signaling, the protein expression of p-MBS and t-MBS was detected by western blotting. The protein was extracted from the peripheral blood samples, and the protein concentration was determined using a bicinchoninic acid (BCA) protein detection kit (Beyotime, Shanghai, China). The proteins were then separated by electrophoresis, transferred to polyvinylidene fluoride (PVDF) membranes, blocked with 5% skimmed milk, and incubated overnight at 4°C with antibody against human MBS, human p-MBS, or human *β*-actin (Santa Cruz, USA). The membranes were then incubated with secondary antibody, and the proteins were visualized using enhanced chemiluminescence reagent (Beyotime). Finally, a ChemiDoc™ XRS + Imaging System (Bio-Rad, USA) was used to take photos of the membranes, and the results were quantified to calculate the p-MBS/t-MBS ratio in each sample.

### 2.9. Statistical Analysis

The data were processed in SPSS v19.0 software. The data are expressed as mean ± standard deviation. The two groups were compared using paired *t* tests. *P* < 0.05 indicates a significant difference.

## 3. Results

### 3.1. Effect of YHWLP on the Clinical Symptom Score of Patients with CPHD

**Figure 1:**
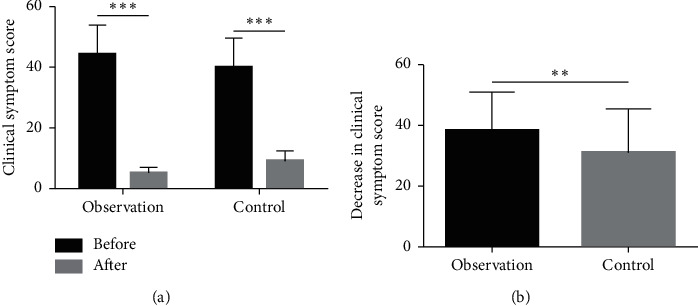
Effect of YHWLP on the clinical symptom score of patients with CPHD. (a) Comparison of clinical symptom score after treatment vs. baseline in each group; (b) comparison of decrease in clinical symptom score (relative to baseline) between the two groups. Values are mean ± standard deviation. ^∗∗^*P* < 0.01 and ^∗∗∗^*P* < 0.001.

There was no significant difference in the baseline clinical symptom score between the two groups. After treatment, the clinical symptom score was significantly lower than at baseline in each group (*P* < 0.001) (Figure 1(a)). The YHWLP group had a significantly greater decrease in the clinical symptom score relative to the control group (Figure 1(b), *P* < 0.01).

### 3.2. Effects of YHWLP on Pulmonary Function in Patients with CPHD

**Figure 2:**
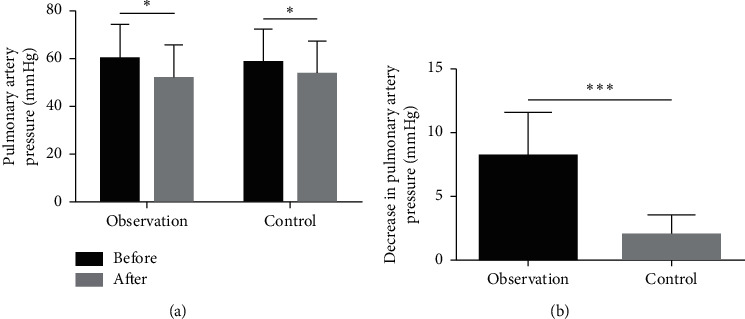
Effect of YHWLP on pulmonary artery pressure in patients with CPHD. (a) Comparison of pulmonary artery pressure after treatment vs. baseline in each group; (b) comparison of decrease in pulmonary artery pressure (relative to baseline) between the two groups. Values are mean ± standard deviation. ^*∗*^*P* < 0.05 and ^∗∗∗^*P* < 0.001.

There was no significant difference in the baseline pulmonary artery pressure between the two groups. After treatment, the pulmonary artery pressure was significantly lower than at baseline in each group (Figure 2(a), *P* < 0.05). The YHWLP group had a significantly greater decrease in pulmonary artery pressure relative to the control group (Figure 2(b), *P* < 0.001). In addition, as shown in Table 1, there were no significant differences in the baseline pulmonary function indicators between the two groups. After treatment, FEV1, FVC, FEV1/FVC, and FEV1% were significantly higher than at baseline in each group (*P* < 0.05). The YHWLP group had a significantly greater increase in each index relative to the control group (Table 2, *P* < 0.05).

### 3.3. Effects of YHWLP on PT, aPTT, Fbg, and D-D in Peripheral Blood of Patients with CPHD

There were no significant differences in the baseline PT, aPTT, Fbg, and D-D between the two groups. After treatment, these values were significantly lower than at baseline in each group (*P* < 0.05) (Table 3). The YHWLP group had significantly greater improvements in these values relative to the control group (*P* < 0.05) (Table 4).

### 3.4. Effect of YHWLP on the p-MBS/t-MBS Ratio in Peripheral Blood of Patients with CPHD

**Figure 3:**
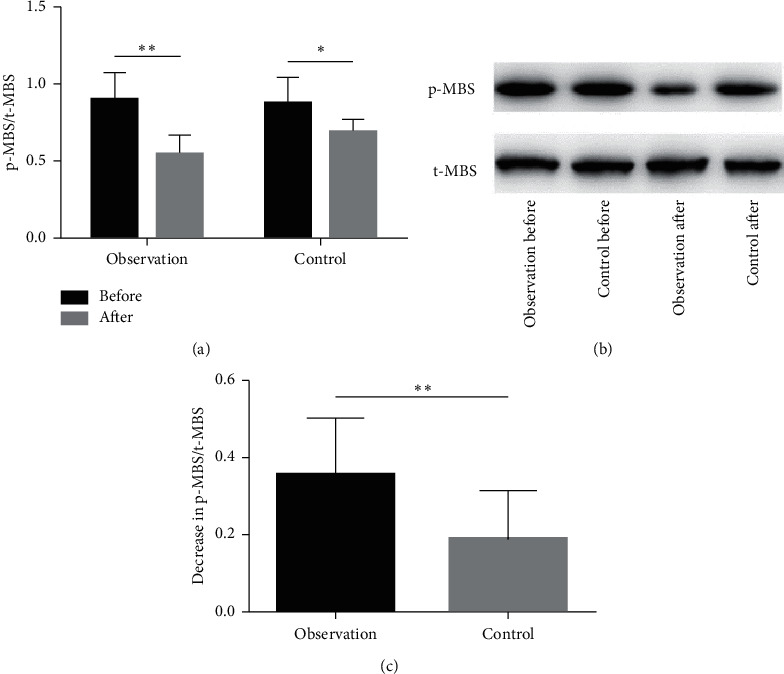
Effect of YHWLP on the p-MBS/t-MBS ratio in peripheral blood of patients with CPHD. (a) Comparison of p-MBS/t-MBS ratio after treatment vs. baseline in each group. (b) Western blotting images. (c) Comparison of decrease in p-MBS/t-MBS ratio (relative to baseline) between the two groups. Values are mean ± standard deviation. ^*∗*^*P* < 0.05 and ^∗∗^*P* < 0.01.

There was no significant difference in the baseline p-MBS/t-MBS ratio between the two groups. After treatment, the ratio was significantly lower than at baseline in each group, indicating RhoA/ROCK signaling inhibition (Figures 3(a) and 3(b), *P* < 0.05). The YHWLP group had a significantly greater decrease in p-MBS/t-MBS relative to the control group (Figure 3(c), *P* < 0.01).

## 4. Discussion

CPHD is a type of heart disease caused by decreased lung function or lung failure, which has a high incidence in China and increases with age [24]. It mainly involves pulmonary hypertension and heart disease. Pulmonary hypertension is a prerequisite for pulmonary heart disease, and heart disease is the final manifestation [25]. In this study, we explored the therapeutic effect and mechanism of YHWLP on COPD-induced CPHD. We found that YHWLP effectively improved pulmonary function indexes, reduced pulmonary hypertension, improved coagulation function indexes, and reduced the p-MBS/t-MBS ratio in peripheral blood. Therefore, YHWLP may play a therapeutic role, which involves inhibiting the RhoA/ROCK signaling pathway.

TCM theory states that there are symptoms of “Yangqi Liangxu, Shuiting Luoyu” in patients with CPHD in the compensatory stage. In addition to cough and puffiness, the patients often have cyanosis of the lips, light/dark swollen tongue, weak and bounding pulse or astringent pulse, and many additional blood stasis symptoms. In recent years, a growing number of studies have confirmed that TCM can have significant effects on CPHD. For example, Gen and Jiang [26] used TCM (ground Dangshen, *Astragalus*, etc.) to tonify the kidneys and benefit the lungs of 46 patients with CPHD. The symptoms and blood flow of the patients were significantly improved. Chen et al. [27] assessed the clinical effects of Linggui Zhugan decoction and Tingli Dazao Xiefei decoction as a representative Wenyang Huayin prescription for CPHD patients, and the blood flow indexes and right ventricular function were significantly improved. Therefore, Prof. Shi Suofang of our research group proposed assessing the clinical effects of YHWLP on CPHD, based on the disease characteristics.

After treating the patients with YHWLP plus Western medicine or Western medicine only, we found that the clinical symptom scores in both groups were significantly improved relative to baseline. However, the degree of improvement was significantly better in the YHWLP group relative to the control group. Moreover, several studies have shown that the pathogenesis of COPD-induced CPHD may involve respiratory dysfunction caused by COPD, which further leads to hypoxemia, pulmonary vasoconstriction, and structural remodeling. This eventually increases the pulmonary vascular resistance and causes pulmonary hypertension [15, 28–30]. Therefore, effectively reducing pulmonary hypertension is the key to treating CPHD.

In this study, we found that both YHWLP + Western medicine and Western medicine alone significantly reduced pulmonary hypertension, but the effect of the former was significantly greater. In addition, long-term hypoxia and carbon dioxide retention in CPHD patients can aggravate the abnormal lung structure and function. As a result, the inflammatory secretions cannot be easily removed, the respiratory tract's immunity against pathogens is decreased, and the patient becomes susceptible to infection [31]. Our results showed that each group had significantly improved pulmonary ventilation function (FEV1, FVC, FEV1/FVC, and FEV1%). However, the YHWLP group had significantly greater increases in these indicators relative to the control group. Therefore, our results confirm that YHWLP + Western medicine is superior to Western medicine alone for treating CPHD.

Previous studies have shown that, in CPHD patients, abnormal changes in the lung microenvironment may lead to vascular endothelial cell dysfunction, causing abnormal release of many coagulation mediators and inflammatory factors. This eventually leads to abnormal coagulation and issues with the fibrinolysis system [1, 32–34]. Thus, we also explored the effects of YHWLP on PT, aPTT, Fbg, and D-dimer in CPHD patients. PT is a common index for assessing coagulation function, and it depends on the levels of coagulation factors produced in the liver [35]. aPTT is a sensitive index of coagulation function. Fbg plays an important role in platelet aggregation. With improvements in blood coagulation function, PT and aPTT decrease, while Fbg increases [36]. D-dimer is a degradation product of fibrin, and the D-dimer level significantly increases as the severity of pulmonary infection increases. Assessing the D-dimer level is helpful to identify thrombotic diseases early and guide the use of follow-up medication [37]. Interestingly, blood coagulation function improved similar to the improvement in lung function in the YHWLP group, with greater improvements than those induced by Western medicine alone.

RhoA, a component of the RhoA/ROCK signaling pathway, belongs to the Ras superfamily. This pathway is closely related to the occurrence and development of pulmonary diseases such as pulmonary hypertension, pulmonary fibrosis, and lung cancer [38–40]. The activation of this pathway is positively correlated with pulmonary hypertension and significantly increased by inflammation. In addition, ROCK inhibitors (fasudil, Y27632, etc.) effectively reduce the pulmonary arterial pressure in rats with pulmonary hypertension [41]. Therefore, effective inhibition of the RhoA/ROCK signaling pathway may be the key to treating CPHD. In this study, we explored the effect of YHWLP regarding RhoA/ROCK signaling inhibition for the first time. As directly assessing the expression of proteins in the RhoA/ROCK signaling pathway would require fresh lung tissue samples, we used an indirect assessment method. The degree of phosphorylation of MBS, a substrate of RhoA-kinase, indirectly reflects the activation of the Rho/ROCK signaling pathway in the lungs of CPHD patients. The results showed that, after treatment, p-MBS/t-MBS was significantly lower relative to baseline in each group. The YHWLP group had a significantly greater decrease in p-MBS/t-MBS relative to the control group. Thus, YHWLP may exert its therapeutic effect by inhibiting the RhoA/ROCK signaling pathway.

In conclusion, the results of this study showed that YHWLP can effectively improve pulmonary function indexes, reduce pulmonary hypertension, and improve coagulation function indexes in COPD-induced CPHD patients. The mechanism may involve inhibiting the RhoA/ROCK signaling pathway. In a follow-up study, we will further explore the mechanism by which YHWLP improves CPHD symptoms via inhibition of the RhoA/ROCK signaling pathway and further verify the potential of YHWLP for treating CPHD.

## Figures and Tables

**Table 1:** Comparison of pulmonary function (after treatment vs. baseline) in each group.

Group		FEV1	FVC	FEV1/FVC	FEV1%
YHWLP (*n* = 40)	Baseline	1.37 ± 0.45	2.51 ± 0.58	54.20 ± 7.84	56.95 ± 8.82
	After	1.76 ± 0.54^*∗∗*^	2.90 ± 0.65^*∗∗*^	60.55 ± 6.19^*∗∗*^	63.06 ± 9.20^*∗*^
Control (*n* = 40)	Baseline	1.44 ± 0.45	2.59 ± 0.57	54.46 ± 6.69	57.75 ± 9.68
	After	1.50 ± 0.43^*∗*^	2.70 ± 0.52^*∗*^	56.38 ± 6.73	63.56 ± 8.77^*∗*^

Notes: FEV1, forced expiratory volume in the first second; FVC, forced vital capacity; FEV1%, FEV1 as a percentage of predicted value. Values are mean ± standard deviation. ^*∗*^*P* < 0.05, ^∗∗^*P* < 0.01.

**Table 2:** Comparison of increases in pulmonary function (relative to baseline) between the two groups.

Group	FEV1	FVC	FEV1/FVC	FEV1%
YHWLP (*n* = 40)	0.39 ± 0.15^*∗∗*^	0.39 ± 0.28^*∗*^	6.35 ± 2.74^*∗∗∗*^	6.11 ± 3.49^*∗*^
Control (*n* = 40)	0.04 ± 0.01	0.21 ± 0.11	1.56 ± 0.52	4.72 ± 5.10

Notes: FEV1, forced expiratory volume in the first second; FVC, forced vital capacity; FEV1%, FEV1 as a percentage of predicted value. Values are mean ± standard deviation. ^*∗*^*P* < 0.05, ^∗∗^*P* < 0.01, ^∗∗∗^*P* < 0.001.

**Table 3:** Comparison of PT, aPTT, Fbg, and D-dimer (after treatment vs. baseline) in each group.

Group		PT (s)	aPTT (s)	Fbg (g/L)	D-D (mg/L)
YHWLP (*n* = 40)	Baseline	14.64 ± 2.36	42.21 ± 3.77	5.59 ± 0.74	5.01 ± 1.24
	After	11.69 ± 1.79^*∗*^	37.96 ± 2.99^*∗*^	2.93 ± 1.33^*∗∗*^	1.92 ± 0.98^*∗∗*^
Control (*n* = 40)	Baseline	13.73 ± 1.55	40.79 ± 3.11	5.38 ± 0.69	5.42 ± 0.79
	After	12.28 ± 1.33^*∗*^	38.67 ± 3.29^*∗*^	4.10 ± 0.87^*∗*^	3.89 ± 1.43^*∗*^

Notes: PT, prothrombin time; aPTT, activated partial thromboplastin time; Fbg, fibrinogen; D-D, D-dimer. Values are mean ± standard deviation. ^*∗*^*P* < 0.05, ^∗∗^*P* < 0.01.

**Table 4:** Comparison of decreases in PT, aPTT, Fbg, and D-dimer (relative to baseline) between the two groups.

Group	PT (s)	aPTT (s)	Fbg (g/L)	D-D (mg/L)
YHWLP (*n* = 40)	2.96 ± 1.72^*∗∗*^	4.25 ± 2.44^*∗*^	2.67 ± 1.61^*∗∗*^	3.08 ± 1.52^*∗∗*^
Control (*n* = 40)	1.45 ± 0.58	2.12 ± 0.64	1.28 ± 0.36	1.53 ± 1.69

Notes: PT, prothrombin time; aPTT, activated partial thromboplastin time; Fbg, fibrinogen; D-D, D-dimer. Values are mean ± standard deviation. ^*∗*^*P* < 0.05, ^∗∗^*P* < 0.01.

## Data Availability

The datasets used and/or analyzed during the current study are available from the corresponding author on reasonable request.

## Supplementary Materials

Supplementary Materials Figure S1: Result of concentrated decoction of YHWLP by HPLC. Notes: 1. Mulberry glycoside a; 2. Paeoniflorin; 3. *Codonopsis* alkynoside; and 4. Glycyrrhizic acid. Figure S2: Result of granules of YHWLP by HPLC. Notes: 1. Mulberry glycoside a; 2. Paeoniflorin; 3. *Codonopsis* alkynoside; and 4. Glycyrrhizic acid.
