# Supplementary material for: “Yiqi Huayu, Wenyang Lishui” Prescription (YHWLP) Improves the Symptoms of Chronic Obstructive Pulmonary Disease-Induced Chronic Pulmonary Heart Disease by Inhibiting the RhoA/ROCK Signaling Pathway
Source: Evid Based Complement Alternat Med. 2021 Oct 26;2021:6636426. doi: 10.1155/2021/6636426 (PMC8563114; doi:10.1155/2021/6636426)
Supplement: Supplementary Materials — Figure S1: Result of concentrated decoction of YHWLP by HPLC. Notes: 1. Mulberry glycoside a; 2. Paeoniflorin; 3. Codonopsis alkynoside; and 4. Glycyrrhizic acid. Figure S2: Result of granules of YHWLP by HPLC. Notes: 1. Mulberry glycoside a; 2. Paeoniflorin; 3. Codonopsis alkynoside; and 4. Glycyrrhizic acid. [file 6636426.f1.docx]

Figure S1 Result of concentrated decoction of YHWLP by HPLC


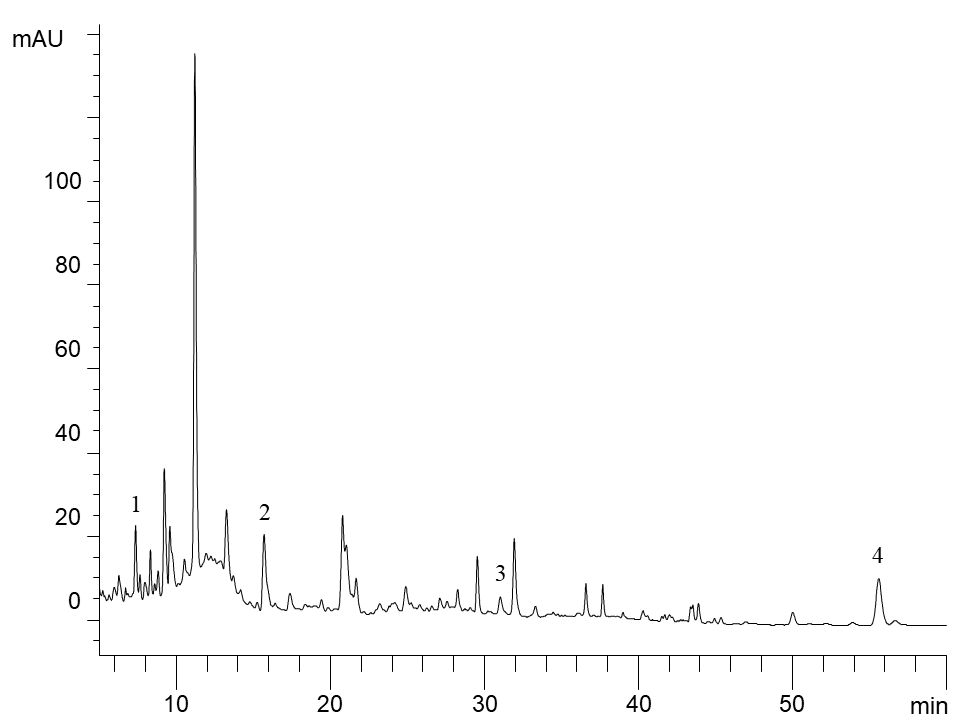


Notes: 1. Mulberry glycoside a; 2. Paeoniflorin; 3. Codonopsis alkynoside; 4. Glycyrrhizic acid

Figure S2 Result of granules of YHWLP by HPLC

**
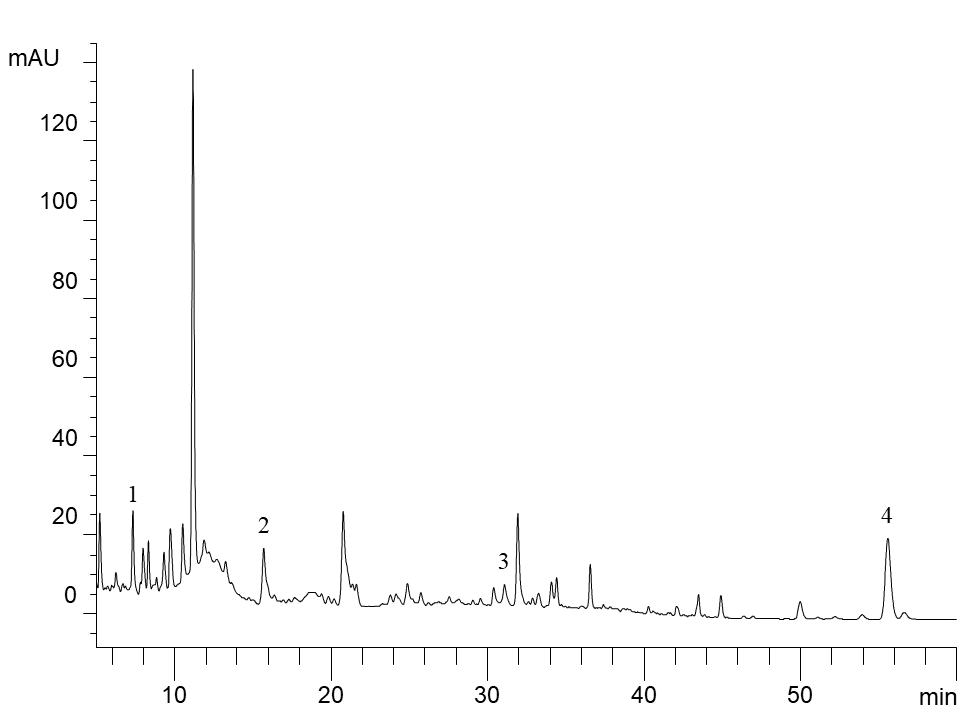
**

**Notes: 1. Mulberry glycoside a; 2. Paeoniflorin; 3. Codonopsis alkynoside; 4. Glycyrrhizic acid**
